# Successful treatment of submassive pulmonary embolism with right ventricle strain using ultrasound-assisted thrombolysis with urokinase: a case report

**DOI:** 10.1007/s00392-023-02191-7

**Published:** 2023-04-09

**Authors:** Hani Al-Terki, Michael Gotzmann, Adnan Labedi, Andreas Mügge

**Affiliations:** Katholisches Klinikum Bochum Sankt Josef-Hospital, Bochum, Germany

## Sirs

Ultrasound-assisted local thrombolysis (USAT) is an effective and safe treatment modality for intermediate–high-risk patients with acute pulmonary embolism (PE) [1]. USAT is superior to anticoagulation alone in terms of improving RV dysfunction, but requires significantly less dosage of thrombolytic substances as compared to systemic thrombolysis [2]. The thrombolytic substance used for USAT is most frequently recombinant tissue-type plasminogen activator (rt-PA); optimal dosage and duration of delivery have been investigated by the OPTALYSE trial [3]. Actually, there is a lack of supply of rt-PA (Alteplase®, Boehringer Ingelheim). We present a case of successful treatment of an 83-year-old patient with bilateral intermediate–high-risk PE with USAT in combination with urokinase (UK) instead of rt-PA.

An 83-year-old man (170 cm, 73 kg, and BMI 25 kg/m^2^) was admitted to the hospital with progressive dyspnoea (NYHA IV), tachypnoea (35 breaths/min), and tachycardia (119 beats/min). A previous echocardiographic examination a week ago revealed normal biventricular function and normal pulmonary pressure (PAPs 26 mmHg, TAPSE 28 mm, and EF 55%).

**Fig. 1:**
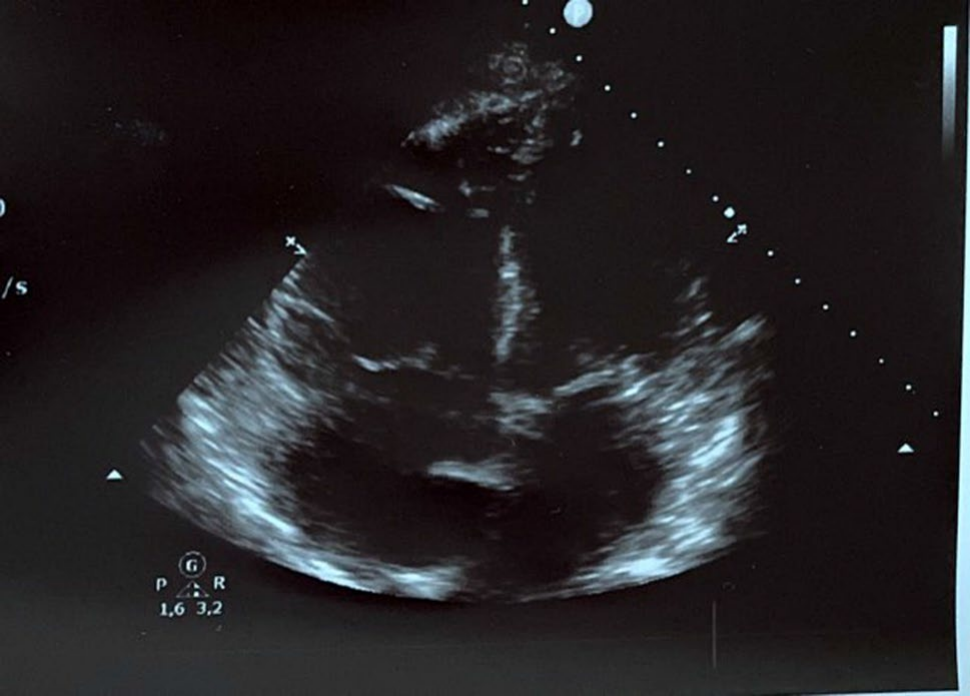
Echocardiography at admission showing a right ventricle dilatation

**Fig. 2:**
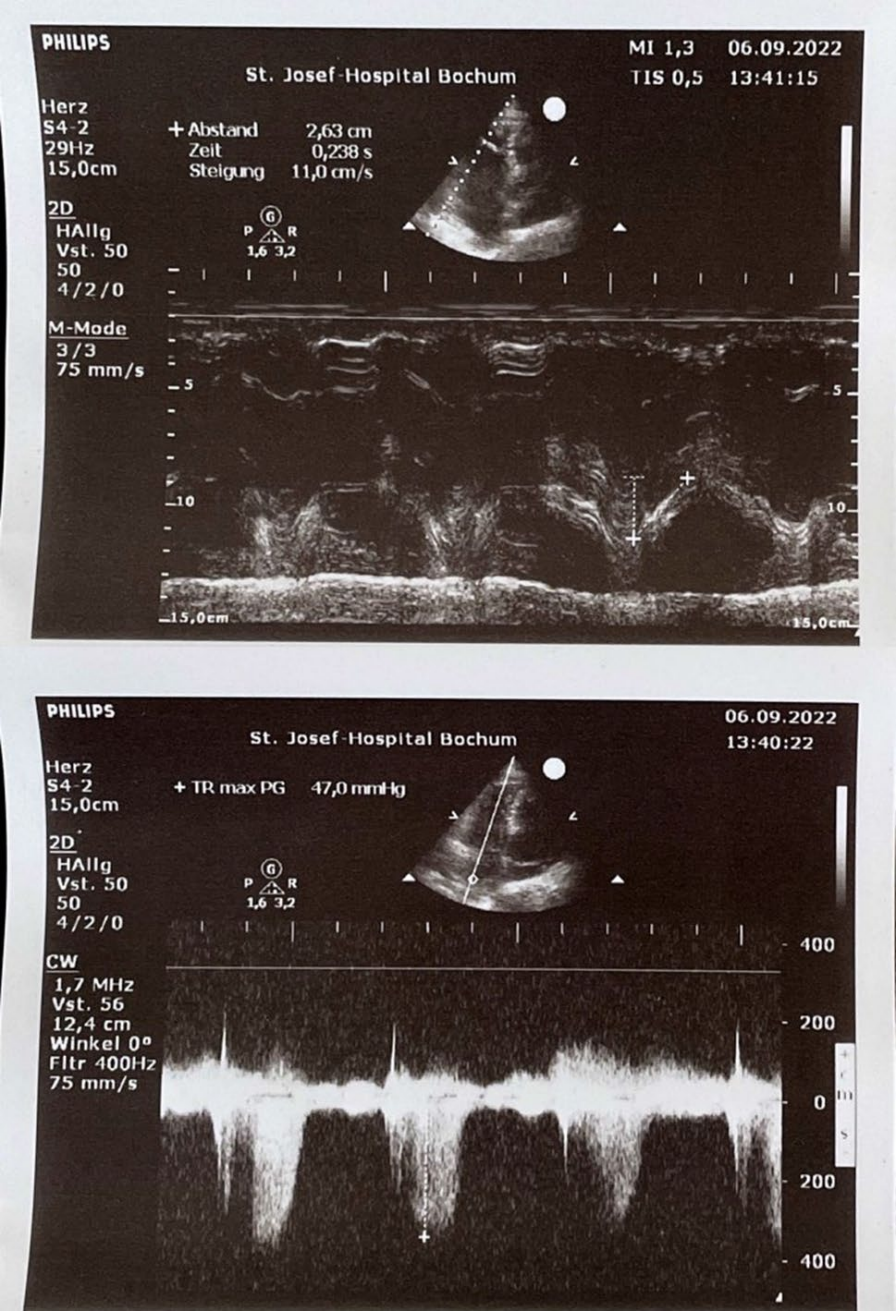
Echocardiography after therapy showing improved right ventricle function

**Fig. 3:**
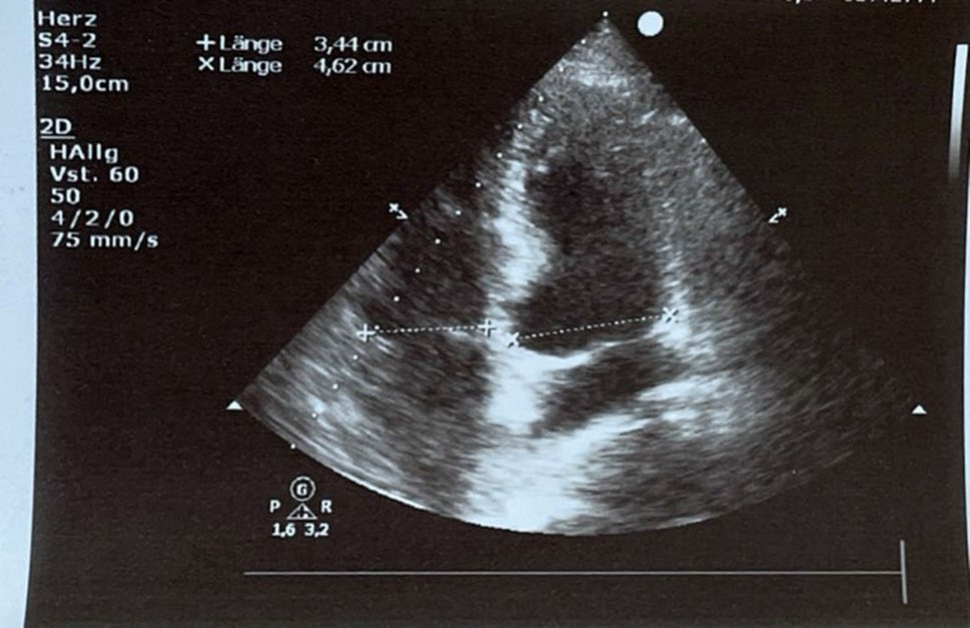
Echocardiography after therapy showing a decrease in the systolic pulmonary artery pressure

Due to the respiratory distress at this presentation, continuous positive airway pressure (CPAP) therapy was initiated. Arterial blood gas analysis under PEEP of 6 mmHg and FiO_2_ of 80% revealed a pH of 7.46; *p*O_2_ 70 mmHg; *p*CO_2_ 41 mmHg, and bicarbonate of 29.4 mmol/L. Laboratory results revealed a d-dimer level of > 20 μg/mL (normal < 0.5 μg/ml), elevated high-sensitive troponin T level of 0.08 ng/mL (normal < 0.01 ng/mL) and elevated NT-proBNP of 2105 pg/mL (normal < 450 pg/mL). A chest X-ray did not show significant findings. However, transthoracic echocardiography revealed a massive dilatation of the right ventricle (RV diameter = 44 mm, RV/LV ratio = 1.25, Fig. 1) with a flattening of the interventricular septum. Systolic pulmonary artery peak pressure was 77 mmHg. Computed tomography revealed a pulmonary thromboembolism in both pulmonary arteries. The patient was admitted to the intensive-care unit, and intravenous heparin therapy was initiated. The respiratory situation deteriorated, and the cPAP therapy was maximally escalated. Despite a FiO_2_ of 100%, oxygen saturation did not reach 90%. Arterial blood gas revealed a *p*O_2_ of 56 mmHg and a *p*CO_2_ of 39 mmHg. Blood pressure remained stable (RRsys > 120 mmHg), but tachycardia did not improve. In view of the elevated sPESI score (2 points), the right-ventricular strain, the elevated troponin and NT-proBNP, and the deterioration under anticoagulation, we decided to use USAT with EKOS (Boston Scientific, Marlborough, MA). Two EKOS catheters were inserted into the pulmonary artery (PA). The invasive PA pressure was 76/48 (57) mmHg. Due to the delivery bottlenecks of rt-PA, we used urokinase (Urokinase, medac, Hamburg, Germany). For a period of 10 h, 30,000 units per hour were given for each catheter. During therapy, the PA pressure dropped to 34/23 (28) mmHg, and NT-proBNP to 1831 pg/mL next day (Table 1). The patient improved quickly. The cPAP ventilation could be deescalated, and switched finally to a O_2_ nasal tube (2 L). The echocardiography revealed RV/LV ratio of 0.7 and estimated PAPs of 45 mmHg (Figs. 2, 3). No complications were noticed.

**Table 1:** Comparison between the value before and after the therapy

	Before therapy	After therapy
RVEDD	44	33
RV/LV ratio	1,25	0.7
PAPs on echo	77	45
PA pressure, invasive	76/48 (57) mmHg	34/23 (28) mmHg
NT-proBNP	2105 pg/mL	1631 pg/mL

The patient could be transferred to the general ward with the recommendation, to continue the oral therapy with 10 mg of apixaban twice daily over 7 days followed by 5 mg twice daily and for a total of 6 months.

Pulmonary embolism is the third leading cause of cardiovascular mortality. The technological landscape for the management of acute intermediate- and high-risk PE is rapidly evolving. Patients with large PE and RV strains, even if normotensive, are at high risk of in-hospital and latent mortality [4]. Thrombolytic therapy improves pulmonary obstruction, PAPs, and pulmonary vascular resistance in patients with PE when compared to heparin alone [5]. These improvements are accompanied by a reduction in RV dilation on echocardiography.

The effectiveness and safety of systemic fibrinolysis in intermediate PE patients who are still normotensive were investigated by the PEITHO Trial [6]. The study compared the use of anticoagulation and systemic fibrinolysis vs. anticoagulation alone in 1005 patients. Systemic fibrinolysis significantly reduced death and hemodynamic decompensation by 56%, albeit at the expense of a fourfold increase in the rate of severe extracranial bleeding (6.3% vs 1.2%) and a 12-fold increase in the rate of intracranial bleeding hemorrhagic strokes (2.4% vs 0.2%). As a consequence, the ESC guidelines do not recommend the routine use of systemic thrombolysis in all PE patients.

Alternatively to systemic fibrinolysis, a catheter-directed local thrombolytic therapy and thrombectomy can be considered [7]. USAT with the EKOS system combines local delivery of thrombolytic substances together with ultrasound, which increases the permeability of the thrombus to thrombolytic drug [2]. In clinical studies, USAT leads to an improvement in right-ventricular pump function, RV/LV ratio, and to a decrease in pulmonary artery pressure in PE patients, with a low rate of procedural complications [1, 2, 8]. Of note, all these studies were performed using thrombolysis with rt-PA. Data about the use of UK in combination with EKOS do not exist.

Regarding PE, UK has been used in different settings. In the Pilot Trial, effectiveness and safety of a low-dose UK regimen were compared with alteplase in patients with an intermediate–high-risk PE. UK was administered with a dose of 10,000 U/kg once a day for 7 days. The rt-PA group was given 50 mg of alteplase via an intravenous injection within 2 h of admission. The efficacy rates were not significantly different. Also, adverse bleeding reactions were higher in the rt-PA group (20.8%) than in the UK group (8.3%), but did not reach significance (*p* = 0.41) [9].

Lin et al. compared 11 patients with massive PE who underwent EKOS with rt-PA (mean dose of 0.86 ± 0.16 mg/h over 17.4 ± 5.23 h) and 15 patients who underwent a catheter-directed-therapy (CDT) with either UK (*n* = 5; total 2.04 ± 0.56 million units delivered over a mean period of 25.3 ± 7.35 h) or rt-PA (*n* = 10; total dose of 25.43 ± 5.27 mg over 26.7 ± 8.64 h). In the CDT group, three major bleeding complications were observed: two groin hematomas and one retroperitoneal hematoma. No bleeding complications were documented in the EKOS group [10].

Cuculi et al. presented the results of a pharmacomechanical approach with prolonged infusion of urokinase after manual aspiration of thrombus using guide catheters on 63 patients. Local thrombolysis was initiated with bolus injection of urokinase of 125,000 U in each microcatheter. The catheters were left in place for at least 24 h with continuous infusion of urokinase (250,000 U/6 h/catheter). The PA pressure dropped significantly from 33 ± 8 to 21 ± 7 mmHg (*p* < 0.001). Five patients died, and nine had major bleeding [11].

In the present case, we combined the use of UK with the EKOS system for the treatment of an intermediate–high risk, elder patient with acute PE. With our protocol, we could significantly improve the hemodynamic and respiratory situation. We thought to communicate this case because of the actual lack of or short-comings in the supply of rt-PA thrombolytic agent, requiring alternative approaches. Since the exact dose of UK with EKOS is not known, we have revised the published articles regarding CDT in submissive PE and UK as a treatment of PE. Lin et.al. found that CDT with 60,000 U/h UK over 25 h has a complete thrombus resolution rate of 50% and a partial thrombolysis of 14% [10]. We assumed that the ultrasound of the EKOS catheter could shorten the duration, so that we started at the half of his dose with closely monitoring of the heart rate and the symptoms. Zhao et al. have achieved a significant clinical improvement and a drop in the PA pressure after no more than 300,000 units UK per CDT [9], so we thought to start at a dose of 30,000 U/h and to continue till 300,000 units for each catheter. The symptoms and the PA pressure has improved, so that we stop the therapy after 10 h. To determine the optimal dose of UK, when USAT are used, further studies are needed.

## Data Availability

The datasets generated and analyzed during the current study are available from the corresponding author on reasonable request.
